# Paternal characteristics associated with maternal periconceptional use of folic acid supplementation

**DOI:** 10.1186/s12884-018-1830-1

**Published:** 2018-05-30

**Authors:** Jan Helge Seglem Mortensen, Nina Øyen, Roy M. Nilsen, Tatiana Fomina, Steinar Tretli, Tone Bjørge

**Affiliations:** 1 Department of Global Public Health and Primary Care, University of Bergen, Kalfarveien 31, N-5018 Bergen, Norway; 2 Department of Obstetrics and Gynecology, Haukeland University Hospital, Bergen, Norway; 3 Center for Medical Genetics and Molecular Medicine, Haukeland University Hospital, Bergen, Norway; 4 Department of Health and Social Sciences, Western Norway University of Applied Sciences, Bergen, Norway; 5 Cancer Registry of Norway, Oslo, Norway

**Keywords:** Pregnancy, Supplement use, Folic acid, Norway

## Abstract

**Background:**

Maternal predictors of folic acid (FA) supplementation use to reduce offspring risk of neural tube defects are well known, while paternal determinants for maternal FA use are less known. Such knowledge is important to increase women’s compliance to recommended periconceptional FA use.

**Methods:**

In a nation-wide study of 683,785 births registered in the Medical Birth Registry of Norway during 1999–2010, the associations between paternal characteristics (age, education, occupation, country of origin) and maternal FA use were estimated by relative risks (RR) with 95% confidence intervals (CI), using log-binomial regression.

**Results:**

Maternal FA use before and during pregnancy (adequate FA use) was found in 16% of the births. The association between paternal age and adequate FA use was inversely U-shaped; adjusted RRs for adequate FA use were 0.35 (95% CI 0.28–0.43) and 0.72 (95% CI 0.71–0.74) for paternal age < 20 and ≥ 40 years, respectively, comparing age 30–34 years. Compulsory education (1–9 years) among fathers was compared to tertiary education; the RR was 0.69 (95% CI 0.68–0.71) for adequate FA use. The lower risk of adequate FA use for paternal compulsory education was present in all categories of maternal education. Occupation classes other than “Higher professionals” were associated with decreased risk of adequate FA use, compared with the reference “Lower professionals”. RR for adequate FA use was 0.58 (95% CI 0.56–0.60) comparing fathers from “Low/middle-income countries” with fathers born in Norway.

**Conclusion:**

Adequate FA use in the periconceptional period was lower when fathers were younger or older than 30–34 years, had shorter education, had manual or self-employed occupations, or originated from low/middle-income countries. Partners may contribute to increase women’s use of periconceptional FA supplementation.

## Background

Folate is necessary in foetal development, and folic acid (FA) supplementation is widely acknowledged to reduce the risk of neural tube defects (NTDs) [1–5]. FA is the synthetic form of the B-vitamin folate, which is essential in the synthesis of DNA, methylation, and DNA repair [6]. Start of FA supplementation prior to conceiving is important in order to reduce the risk of NTDs because the neural tube closes between 21 and 28 days after conception [7].

Randomized clinical trials and non-randomized intervention trials have demonstrated that periconceptional FA use reduces the risk of NTDs [1–3]. Recent studies have reported that FA is associated with protection against other neurodevelopmental disorders and some severe pregnancy complications [8–10]. The protective effect of FA on NTDs has led health authorities in several countries, including Norway, to recommend women to take FA supplements before pregnancy and in early pregnancy [11–13].

Many countries in Europe, including Norway, have performed information campaigns to increase the use of periconceptional FA supplementation among women planning pregnancy [14–16]. Presently there is no mandatory folic acid food fortification in Norway [17]. Official Norwegian guidelines from 1998, states that all women planning their pregnancy should use 0.4 mg FA daily from 1 month before pregnancy and throughout the first 2–3 months of pregnancy to reduce the risk of NTDs [12]. However, the proportion of preconception FA supplementation use in Norway is still too low [18] and by 2015 it was 33% [19].

Previous studies have identified maternal factors associated with inadequate FA in the periconceptional period, such as low maternal age, shorter education, single parenthood, unplanned pregnancy, lower parity, smoking, alcohol use, less physical activity, or originating from a foreign country [15, 16, 20–24].

Since couples tend to exhibit concordant health behaviour’s for dietary intake, smoking, alcohol consumption, physical activity, and body mass index (BMI) [25–27], a woman’s partner may contribute to her use of periconceptional FA supplements. In fact, in an early report from the Norwegian Mother and Child Cohort Study (MoBa), 2000–2003, counting 22,500 women, FA supplements were used more frequently among women with partners with a higher education [22]. However, the study did not assess other paternal factors or combined paternal and maternal factors as to identify women with inadequate FA use.

Taking advantage of the Medical Birth Registry of Norway that to our knowledge is the only national registry with information on periconceptional use of FA supplements [28], we updated parent information with data from national registries to investigate whether paternal factors (age, education, occupation, country of origin) was associated with mothers’ intake of recommended FA in pregnancy.

## Methods

### Data-sources

Maternal FA use before and/or during pregnancy was collected from the Medical Birth Registry of Norway (MBRN) [28]. Paternal and maternal demographic data came from the National Registry (NR). Information on paternal and maternal occupation originated from the Norwegian Labour and Welfare Administration (NAV), and we retrieved paternal and maternal educational data from the Norwegian National Education Database (NUDB) [29].

MBRN is a population-based registry containing information on all births in Norway since 1967 [28]. The registry holds demographic information on the mother and the father, the mother’s health before and during pregnancy, including chronic diseases, information on in vitro fertilization (IVF), complications during pregnancy and delivery as well as information on the infant, including birth defects and other perinatal problems. Midwives and physicians attending the deliveries register the data. Since 1967, there has been mandatory reporting of all live and stillbirths from the 16 gestational week to MBRN.

NR contains demographic information on all residents in Norway since 1960, including the date of birth, country of origin, and the dates of immigration, emigration, or death [30]. NR assigns a unique personal identification number to all individuals born or immigrated to Norway, enabling accurate record linkages.

NAV was established in 2006 after governmental reorganization of the Directorate of Labour in Norway (founded in 1945), and has registered information on occupation, health status, and social benefits of all individuals with residence in Norway since 1992. The Norwegian occupational code system is based on the International Standard Classification of Occupations (ISCO), revised version from 1988 [31].

Since 1970, NUDB holds information on all individuals’ education history from primary school up to doctoral studies in one database [32]. The classification is based on the Norwegian Standard Classification of Education.

### Maternal FA supplement use

We constructed a binary variable for intake of FA supplement use (0.4 mg/day) (regardless of concomitant multivitamin use) registered in the MBRN since December 1998 onwards; adequate FA use (recommended FA supplementation before and during pregnancy), and inadequate FA use (FA supplementation only before pregnancy, or only during pregnancy, or no record of FA use).

MBRN also registers multivitamin use, but our investigation focused on periconceptional FA use as such intake was according to official guidelines.

### Paternal characteristics

We used the following paternal variables in our analyses of adequate FA supplementation; paternal age (< 20, 20–24, 25–29, 30–34, 35–39, 40+ years); education (Compulsory (1–9 years), Intermediate (10–12 years), Tertiary (13–19 years)); occupation according to the class scheme of Erikson, Goldthorpe, and Portocarero (I Higher professionals, II Lower professionals, IIIa Higher routine, IIIb Lower routine, IV Other self-employed workers, V Technicians, VI Skilled, VII Semiskilled and unskilled, VIIb Agricultural, Unclassified) (EGP) [33]; and country of origin according to the classification by World Health Organization, Health statistics and information systems, Estimates for 2000–2012 (Norway, High income countries, Low/middle-income countries) [34].

### Covariates

We used directed acyclic graphs (DAGs) and subject-matter knowledge to select a minimally sufficient adjustment set of variables that identify the unconfounded association of paternal characteristics on adequate maternal FA supplementation use [35–37].

The potential confounders of the paternal characteristics and maternal FA use relationship included year of childbirth (continuous), paternal age (< 20, 20–24, 25–29, 30–34, 35–39, 40+ years), education (Compulsory, Intermediate, Tertiary), or country of origin (Norway, High-income countries, Low/middle-income countries).

Furthermore, we included maternal age (< 20, 20–24, 25–29, 30–34, 35–39, 40+ years), maternal education (Compulsory, Intermediate, Tertiary), and maternal country of origin (Norway, High-income countries, Low/middle-income countries) as possible confounders of the associations between paternal age, education, or country of origin, and maternal adequate FA use.

Maternal smoking was not included in the final models because smoking was not considered a confounder of the association of paternal characteristics on maternal FA supplementation use [38].

### Study population

During 1999–2010, 716,021 births were registered in MBRN. We excluded births (induced abortions) without information on FA or multivitamin supplementation use (2519) and births without maternal identification number (4091). For multiple births, we included data for the first birth and excluded 12,927 next born individuals. Among the remaining 696,484 births, we excluded 12,699 births without paternal identification number, leaving 683,785 live births and stillbirths for analyses.

### Statistical analysis

Associations between paternal characteristics (age, education, occupation, country of origin) and maternal FA use were estimated as relative risks (RRs) with 95% confidence intervals (CIs) by log-binomial regression, using the log-link function in Stata version 15 [39]. The 95% CIs were based on robust variance estimation with the sandwich estimator to correct for the intra-individual correlation in women with more than one pregnancy during the study period [40]. Births with missing data on covariates were excluded from the analyses. *P*-values for overall difference between the categories of paternal characteristics were calculated using likelihood ratio tests. We evaluated and tested the potential effect modification of the association between paternal education and maternal FA use by stratification and likelihood ratio test.

## Results

Our study included 683,785 births during 1999–2010. Table 1 presents the characteristics of the parents. The median ages of the fathers and mothers at childbirth were 33 and 30 years, respectively. For about 41% of the births, the mothers were primiparous, and about 2% of the births were conceived after in vitro fertilization (IVF). The majority of the births were of Norwegian-born parents (84% of the fathers and 83% of the mothers). For about 34% of the births, the fathers had tertiary education, and for about 19% of the births, the fathers had compulsory education only. The paternal educational level varied by his country of origin. Fathers originating from low/middle-income countries generally had lower educational level compared to fathers originating from Norway and other high-income countries (not shown). Occupation classified as “Lower professionals,” accounted for 22% of all the births. For about 14% of the births, the women smoked daily at the start of pregnancy, about 3% smoked intermittently, and 67% did not smoke. Nearly 17% of the smoking data were missing.

**Table 1:** Paternal and maternal characteristics in 683,785 births in Norway, 1999–2010

	**Births**			
	Fathers	%	Mothers	%
*Number of births*	683,785	100.0	683,785	100.0
*Age*				
< 20	4401	0.6	15,464	2.3
20–24	48,448	7.1	100,016	14.6
25–29	162,671	23.8	223,480	32.7
30–34	235,401	34.4	228,203	33.4
35–39	151,540	22.2	99,727	14.6
40+	81,324	11.9	16,895	2.5
*Education*				
Compulsory education (1–9 years)	130,953	19.2	125,479	18.4
Intermediate (10–12 years)	302,384	44.2	230,320	33.7
Tertiary education (13–19 years)	229,818	33.6	298,036	43.6
Missing data	20,630	3.0	29,950	4.4
*Occupational class* ^*a*^				
I Higher professionals	86,635	12.7	50,650	7.4
II Lower professionals	152,781	22.3	122,804	18.0
IIIa Higher routine	77,540	11.3	197,174	28.8
IIIb Lower routine	40,070	5.9	114,795	16.8
IV Other self-employed workers	358	0.1	119	0.0
V Technicians	5550	0.8	1492	0.2
VI Skilled	108,755	15.9	15,961	2.3
VII Semiskilled and unskilled	111,584	16.3	73,994	10.8
VIIb Agricultural	7663	1.1	2720	0.4
Unclassified	52,824	7.7	51,264	7.5
Missing data	40,025	5.9	52,812	7.7
*Country of origin* ^*b*^				
Norway	574,602	84.0	567,241	83.0
High income countries	33,487	4.9	30,920	4.5
Low/middle-income countries	75,497	11.0	85,597	12.5
Missing data	199	0.0	27	0.0
*Marital status*				
Unmarried			37,057	5.4
Married/Partnership			634,283	92.8
Divorced			3417	0.5
Missing data			9028	1.3
In vitro *fertilization (IVF)*				
No			669,024	97.8
Yes			14,761	2.2
*Birth order*				
1			280,178	41.0
2			244,532	35.8
≥3			159,075	23.3
*Maternal chronic disease* ^*c*^				
No			623,817	91.2
Yes			59,968	8.8
*Maternal smoking before pregnancy*				
Non-smoker			456,797	66.8
Intermittent			18,518	2.7
Daily			93,662	13.7
Missing data			114,808	16.8
*Maternal folic acid use in pregnancy*				
No use			371,820	54.4
Only before			8930	1.3
Only during			192,169	28.1
Before and during			110,866	16.2

^a^Categorized according to the class scheme of Erikson, Goldthorpe and Portocarero (EGP) [33]

^b^Categorized according to the classification by World Health Organization, Health statistics and information systems, Estimates for 2000–2012 [34]

^c^Asthma, hypertension, kidney disease, chronic urinary infection, rheumatoid arthritis, heart disease, epilepsy, diabetes mellitus (type I or II), and thyroid disease

For about 16% of all births in the study population, the mothers were assigned to the category adequate FA supplementation users. However, during 1999 through 2010, the proportion of adequate FA supplementation use increased from 4% at the start of the study period (1999) to 26% in 2010.

Table 2 presents crude and adjusted RRs for adequate maternal periconceptional FA use by paternal variables (determinants). Adjusted analyses showed an inverse “U-shaped” relationship between paternal age and adequate maternal FA supplement use where the smallest RRs were found for paternal age below 20 years (RR 0.35 (95% CI 0.28–0.43)), 20–24 years (RR 0.68 (95% CI 0.66–0.71)), and 40 years and above (RR 0.72 (95% CI 0.71–0.74)) compared to paternal age 30–34 years. Paternal compulsory education was associated with reduced risk of adequate FA use (RR 0.69 (95% CI 0.68–0.71)) compared to paternal tertiary education. All paternal occupation classes were associated with reduced risk of adequate FA use except for “I Higher professionals”, when compared to “II Lower professionals”, in particular “VII Semiskilled and unskilled” (RR 0.75 (95% CI 0.73–0.76)), and “VIIb Agricultural” (RR 0.73 (95% CI 0.69–0.78)).

**Table 2:** Relative risks (RRs) with 95% confidence intervals (95% CIs) of adequate maternal periconceptional folic acid supplement use (before and during pregnancy) by paternal characteristics, in 683,785 births, Norway, 1999–2010

	Folic acid supplementation use		Unadjusted		Adjusted for paternal factors ^a b^		Further adjusted for maternal factors ^a c^	
Characteristics	Yes	No	RR*	95% CI	RR*	95% CI	RR*	95% CI
*Paternal age (years)*								
< 20	86	4315	0.11	0.09–0.13	0.10	0.08–0.13	0.35	0.28–0.43
20–24	3325	45,123	0.37	0.36–0.39	0.37	0.36–0.38	0.68	0.66–0.71
25–29	22,886	139,785	0.76	0.75–0.78	0.77	0.76–0.79	0.94	0.93–0.96
30–34	43,348	192,053	1.00	Reference	1.00	Reference	1.00	Reference
35–39	28,488	123,052	1.02	1.01–1.03	0.97	0.96–0.99	0.90	0.89–0.91
40+	12,733	68,591	0.85	0.83–0.87	0.80	0.78–0.81	0.72	0.71–0.74
*Paternal education*								
Compulsory (1–10 years)	11,694	119,259	0.40	0.39–0.40	0.52	0.51–0.53	0.69	0.68–0.71
Intermediate (11–13 years)	45,411	256,973	0.67	0.66–0.67	0.75	0.74–0.76	0.87	0.85–0.88
Tertiary (14–20 years)	51,894	177,924	1.00	Reference	1.00	Reference	1.00	Reference
Missing data	1867	18,763						
*Paternal occupational class* ^*d*^								
I Higher professionals	19,957	66,678	1.05	1.04–1.07	1.05	1.03–1.06		
II Lower professionals	33,366	119,415	1.00	Reference	1.00	Reference		
IIIa Higher routine	12,292	65,248	0.73	0.71–0.74	0.89	0.88–0.91		
IIIb Lower routine	5352	34,718	0.61	0.59–0.63	0.85	0.83–0.87		
IV Other self-employed workers	60	298	0.77	0.61–0.97	0.83	0.65–1.05		
V Technicians	836	4714	0.69	0.65–0.74	0.89	0.84–0.95		
VI Skilled	15,174	93,581	0.64	0.63–0.65	0.84	0.83–0.86		
VII Semiskilled and unskilled	11,643	99,941	0.48	0.47–0.49	0.75	0.73–0.76		
VIIb Agricultural	908	6755	0.54	0.51–0.58	0.73	0.69–0.78		
Unclassified	7575	45,249	0.66	0.64–0.67	0.96	0.94–0.99		
Missing data	3703	36,322						
Paternal country of origin ^e^								
Norway	99,339	475,263	1.00	Reference	1.00	Reference	1.00	Reference
High income countries	6535	26,952	1.13	1.10–1.16	1.06	1.04–1.09	1.06	1.03–1.08
Low-middle-income countries	4975	70,522	0.38	0.37–0.39	0.35	0.34–0.36	0.58	0.56–0.60
Missing data	17	182						

^a^All RRs for adequate folic acid supplementation adjusted for year of childbirth (continuous)

^b^RRs by paternal age, no other adjustment for paternal factors; RRs by paternal education adjusted for paternal age (< 20, 20–24, 25–29, 30–34, 35–39, 40+), paternal country of origin (Norway, high-income countries, low/middle-income countries); RRs by paternal occupation adjusted for paternal age, fathers country of origin, fathers education (compulsory, intermediate, tertiary); and RR by paternal origin of country, no other adjustment for paternal factors

^c^RRs by paternal age, further adjusted for maternal age (< 20, 20–24, 25–29, 30–34, 35–39, 40+); RRs by paternal education, further adjusted for maternal education (compulsory, intermediate, tertiary); RRs by paternal occupation, no further adjustment for maternal factors; RRs by paternal country of origin adjusted for maternal country of origin (Norway, High-income countries, Low/middle-income countries)

^d^Categorized according to the class scheme of Erikson, Goldthorpe and Portocarero (EGP) [33]

^e^Categorized according to the classification by World Health Organization, Health statistics and information systems, Estimates for 2000–2012 [34]

*p-value for difference between categories of paternal characteristics was < 0.001 using likelihood ratio test

Mothers whose children’s father originated from low/middle-income countries had also a reduced risk of adequate FA use (RR 0.58 (95% CI 0.56–0.60)) compared to fathers originating from Norway.

Table 3 presents crude and adjusted RRs with 95% CIs of adequate FA use by maternal and paternal education. Adjusted analyses showed that adequate FA use was less likely in births were fathers had compulsory education, regardless of maternal education. The association of paternal compulsory education and recommended FA use was weakened by increasing level of maternal education. However, even when the mother had tertiary education, the association of compulsory paternal education on adequate maternal FA use was significant (RR 0.75 (95% CI 0.73–0.77)), compared to fathers with tertiary education.

**Table 3:** Relative risks (RRs) with 95% confidence intervals (95% CI) of adequate maternal periconceptional folic acid supplement use (before and during pregnancy) by combining maternal and paternal education in 683,785 pregnancies, Norway, 1999–2010

		Adequate folic acid use		Unadjusted		Adjusted ^a^	
Maternal education	Paternal education	Yes	No	RR*	95% CI	RR*	95% CI
Compulsory education	Compulsory education	2557	48,326	0.46	0.43–0.50	0.53	0.50–0.57
	Intermediate education	4758	52,693	0.77	0.72–0.81	0.76	0.72–0.81
	Tertiary education	1292	10,651	1.00	Reference	1.00	Reference
	Missing information	208	4994				
Intermediate education	Compulsory education	5079	43,708	0.60	0.57–0.62	0.66	0.64–0.68
	Intermediate education	18,055	116,378	0.77	0.75–0.79	0.81	0.79–0.83
	Tertiary education	7477	35,269	1.00	Reference	1.00	Reference
	Missing information	426	3928				
Tertiary education	Compulsory education	3805	17,985	0.70	0.67–0.72	0.75	0.73–0.77
	Intermediate education	22,150	80,373	0.86	0.85–0.87	0.88	0.87–0.89
	Tertiary education	42,541	126,896	1.00	Reference	1.00	Reference
	Missing information	747	3539				
Missing data (maternal education)		28,179	1771				

^a^Adjusted for paternal age (< 20, 20–24, 25–29, 30–34, 35–39, 40+), year of childbirth (1999–2010 (continuous)), paternal country of origin (Norway, high income countries, low/middle income countries), stratified by maternal education

*p values for interaction between maternal and paternal education were calculated by likelihood-ratio tests (unadjusted p value < 0.001; adjusted p value < 0.001)

## Discussion

The present population-based study (683,785 births during 1999–2010) showed that recommended maternal FA use was low among fathers who were young or older at their children’s birth, had achieved shorter education, held a manually or self-employed occupation, or originated from low/middle-income countries. Even among mothers who had achieved higher education, recommended periconceptional maternal FA use was low among less educated fathers.

Several studies have investigated the association between maternal socio-demographic, reproductive, and medical characteristics and adherence to recommended intake of periconceptional FA. A common feature among mothers is that young age, low educational level, low socioeconomic status, unplanned pregnancy, higher parity, smoking, single marital status, and non-western birthplace is the most important determinants for inadequate FA supplementation use [20–23]. Furthermore, maternal chronic diseases and IVF were positively associated with adequate periconceptional FA supplementation use [22, 38].

In Denmark, a cross-sectional study consisting of 22,000 pregnant women (primiparious and multiparous) showed that only 14% of the women used FA as recommended and compliance was positively associated with being primiparous, older than 25 years and non-smoker [21]. Similarly, for about 16% of the births in our study, the mothers had followed the national guidelines of FA use in the study period (1999–2010).

In Norway, a publication from the Norwegian Mother and Child Cohort Study (MoBa), comprising 27% of the births registered in MBRN during 2000–2003, showed similar results to ours [22]. They found a positive association between paternal education and recommended periconceptional FA use. In pregnancies with fathers having university or college education the adjusted relative risk (RR) of periconceptional maternal FA use was 1.4 (95% CI 1.1–1.8) compared to pregnancies with fathers with primary education. However, the association was weaker than for maternal education. When paternal *tertiary* education was compared to paternal *compulsory* education (reference) in our analyses, we found a similar result for adjusted RR of 1.45 (95% CI 1.42–1.48).

Couples who live together share the same environment, social network, financial resources, and to some extent, the same health risk; beneficial or negative to health outcomes depending on the health behaviour of the spouses [25, 27]. Furthermore, a Dutch study of 40,000 individuals aged 25–74 years showed that women seems more affected by their partner’s educational level than men are with regard to healthy behaviour [41].

In accordance with our findings, a cross-sectional household survey conducted in Pakistan (comprising 6266 women), showed that maternal intake of iron and FA supplements was positively associated with the educational status of the mothers’ husband [42].

The association of ethnic background and maternal periconceptional FA use have been studied in Norway and other European countries (Netherlands, Belgium, Ireland and the United Kingdom) [23, 43–46]. These studies show that supplement use is less common among most ethnic minority groups than among the comparison groups. We have similar findings in our study, showing a lower risk of adequate maternal FA use among fathers originating from low/middle-income countries.

The strengths of our study included use of comprehensive data from population-based registries in Norway that assures generalizability of our results, and registration of individual-level information on periconceptional FA intake for all births in Norway since 1999 (except for terminated pregnancies).

Our study had some limitations. Maternal FA intake could have been misclassified; in the beginning of the study period, FA users were underreported to the MBRN [47]. Our results may therefore be somewhat weaker than the true associations. Furthermore, we could not adjust for pregnancy planning, maternal physical activity or maternal use of alcohol [16, 18, 20–24], as these potential confounders/covariates were not available in our dataset. However, a recent longitudinal study during 2014 on men’s pregnancy planning comprising about 800 participants in Sweden, showed that 81% of the pregnancies were planned and the level of paternal education was positively associated with pregnancy planning [48]. Moreover, data from 22,500 mothers in the MoBa study with deliveries recorded in 2000–2003 showed that 78% of the mothers had planned their pregnancy [22]. However, MoBA is not entirely representative of the total pregnant population in Norway, since the participants are somewhat better educated, slightly older at delivery, and with a lower percentage of smokers than the overall pregnant population.

Information about fathers was not available in 12,699 births (2% of all births in the study population) and were excluded from the study population. They represent births with fathers unreported by the pregnant woman or fathers without identification number from the NR. Among the excluded births (missing father information), 10% of the mothers had adequate periconceptional FA supplementation (16% in the study population) with an RR of 0.63 (95% CI 0.60–0.66) for adequate maternal FA use comparing births with unregisterd fathers with births having registered fathers.

Adjusting for maternal confounders (maternal age, education, or country of origin) in our analysis reduced the strength of the associations between paternal determinants (age, education or country of origin) and adequate maternal periconceptional FA use. This suggest that paternal factors are important, but targeting maternal demographic and socioeconomic conditions and other factors related to low use is still important. However, our findings have implications for public health practice. Recent research on men’s birth intentions has shown that 63% of pregnancies were intended (wanted) by the father [49]. Further, our study demonstrates the importance of the partner’s impact on maternal reproductive health and family planning through shared decision-making.

## Conclusions

In conclusion, our study supports the importance of father’s prenatal role in their children’s health. In order to improve maternal periconceptional FA supplementation use, information and knowledge about the importance of FA’s preventive potential needs to be directed to both men and women. Furthermore, our findings show that women having partners originating from low/middle-income countries, partners at age < 30 and > 34 years, having compulsory education only, and having occupations other than “higher professional”, compared to “lower professionals”, are particularly susceptible to low periconceptional FA use. Therefore, campaigns for improved FA supplementation use should focus particularly on these groups.

## Abbreviations

BMI: Body mass index
DAGs: Directed acyclic graphs
IVF: In vitro fertilization
MBRN: Medical Birth Registry of Norway
MoBa: Norwegian Mother and Child Cohort Study
NAV: Norwegian Labour and Welfare Administration
NTDs: Neural tube defects
NUDB: Norwegian National Education Database
